# CPP or Not, That Is the Question: Physicians’ Work With Activating CPPs

**DOI:** 10.1177/10497323211020708

**Published:** 2021-06-07

**Authors:** Siri Christine K. Næss

**Affiliations:** 1 Norwegian University of Science and Technology, Trondheim, Norway

**Keywords:** institutional ethnography, cancer, standardized cancer patient pathways, referral work, primary/specialist care, qualitative interviews, Norway

## Abstract

The Norwegian government has launched a policy titled cancer patient pathways (CPPs), which assigns maximum deadlines to the various phases of the diagnostic investigation. In this article, I examine the starting point of CPPs through the lens of institutional ethnography—that is, how physicians work with the referral of patients in the context of CPPs. Based on qualitative interviews with physicians in both primary and secondary care across Norway (*N* = 37), the findings reveal that the distinction between CPP or not is by no means clear-cut for either primary or specialist physicians. The starting point of CPPs is mediated by the interaction between physicians and patients and how the referral is composed, as well as how and by whom the referral is interpreted, in conjunction with overarching discourses, policies, and guidelines for practice. The findings challenge the notion that all potential cancer patients can and should be equally prioritized.

## Introduction

Despite the absence of a clear connection between a timely cancer diagnosis and survival, there is a growing body of evidence suggesting that early detection and treatment of cancer are likely to influence the prognosis positively (Neal, 2009). Consequently, significant attention is being paid to the importance of accelerating the diagnostic process (Malmström et al., 2018; Rubin et al., 2011; Wilkens et al., 2016). In Norway, as well as Denmark and Sweden, the notion that time can be used as an essential strategy to fight cancer inspired the development of a policy titled cancer patient pathways (CPPs), implemented in 2015. The intention is to improve the quality of cancer care by providing all potential cancer patients with a standardized set of time frames, from suspicion of cancer to diagnosis and the start of treatment. There are 28 CPPs for different types of cancer. The CPPs are anchored in the clinical practice guidelines, which provide recommendations for diagnostic procedures but are concerned with the logistics (Norwegian Directorate of Health, 2016). In this article, I examine how primary and specialist physicians, balancing diverse demands, work with the referral of patients to CPPs.

For most patients, the path to diagnosis starts with noticing symptoms and presenting them to a general practitioner (GP). How the GP responds is decisive for further action (Lyratzopoulos et al., 2012; Macleod et al., 2009). Because knowing when and where to refer a patient is not a clear-cut science, there is great variation in referral practices between physicians (Greenhalgh, 2002; Thorsen et al., 2012). Greenhalgh (2002) points out that clinical decision making is a delicate process whereby the principles of evidence-based medicine merge with the experience-based and intuitive gaze of the physician, which she describes as “the science of intuition” (p. 399). Furthermore, physicians’ referral practice is contextual and relies profoundly on interactions with patients, the way patients present their unique concerns and experiences, and how these are interpreted by physicians in conjunction with national guidelines, previous experience (both their own and their associates in the medical community), and the organizational structures framing their practice (Gabbay & Le May, 2004; Greenhalgh, 2002; Shaw et al., 2005; Thorsen et al., 2012).

This inherent variation in practice, coupled with the tricky nature of cancer symptoms—they are often diffuse and overlap symptoms of other, more benign conditions, which makes it difficult to decide whether it is more appropriate to make a referral or to wait and see how the symptoms progress—makes the transition from primary to specialist care particularly vulnerable to delays in cancer diagnoses (Andersen & Vedsted, 2015; Green et al., 2015; Hamilton, 2010; Hultstrand et al., 2020; Macleod et al., 2009). Studies demonstrate that there is great variety in the number of primary care visits before patients are referred to the hospital for suspected cancer, and thus many patients will consult their GP several times before a referral to specialist assessments is made (Ewing et al., 2018; Lyratzopoulos et al., 2012).

Because referrals of patients for cancer diagnoses are so varied, more knowledge is needed to better understand how this process is actually carried out. In other words, how a reasonable suspicion of cancer is achieved in reality. How do physicians work with the referral of patients to cancer diagnoses, and what regulates their practice? Although GPs play a pivotal role in early diagnoses by promptly referring patients to specialist care, the priority assigned to referrals by health care providers in specialist health care also interferes with the length of time from suspicion to diagnosis (Olesen et al., 2009). This makes researching what happens in the interface between primary and specialist care physicians particularly relevant in the context of policies such as the CPPs, which targets the rapid detection and treatment of cancer. Although some studies have investigated CPPs from the perspectives of health professionals (Delilovic et al., 2019; Hultstrand et al., 2020; Melby & Håland, 2021; Næss & Håland, 2021), there is no study (to my knowledge) targeting the interface between primary care and specialist care as it relates to the starting point of CPPs.

As with other Nordic countries, the Norwegian health care system is predominantly tax financed and provides universal access. All Norwegian citizens have the right to a dedicated primary physician (GP) of their own choosing (Iversen et al., 2016). The GP is the primary starting point for a CPP. GPs may initiate a CPP, but it is the specialist who ultimately decides (based on the referral) whether the patient is assigned to a CPP.

**Figure 1.**
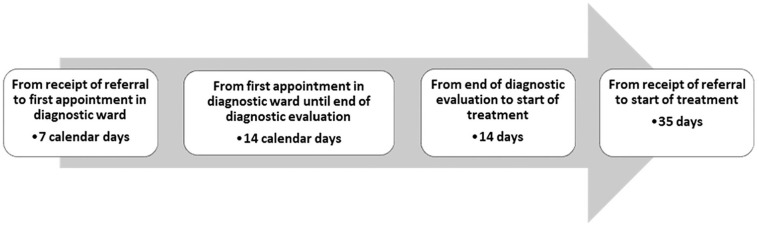
Example of CPP time frames for malignant melanoma. *Note.* CPP = cancer patient pathway.

The CPPs consist of four time frames, as illustrated in Figure 1. The first time frame, which is the focal point of this article, is activated when the hospital receives a referral documenting a “reasonable” suspicion of cancer. In national policy documents, this is depicted as a linear movement with a clear starting point (Norwegian Directorate of Health, 2021). However, I aim to demonstrate that the work involved in starting CPPs is complex, with several overlapping interfaces between primary care and different hospital departments. As the inquiry is guided by institutional ethnography, it illuminates aspects of the broader social organization shaping these work processes. The article aims to contribute to knowledge of the tension between bureaucratic processes articulated through policy documents and what happens when policy documents, such as the CPPs, hit the actualities of clinical practice.

## Theory and Method

### Institutional Ethnography

The study is theoretically and methodologically underpinned by institutional ethnography. Conceived by sociologist Dorothy Smith, institutional ethnography is an approach to inquiry designed to uncover the social organization of people’s activities—what Smith (1987) refers to as “the relations of ruling/ruling relations” (pp. 4–5). The concept of ruling relations anchors institutional ethnography in a power perspective, as it refers to all the social institutions (e.g., government, bureaucracies, laws, financial management, educational institutions, mass media, textual discourses) that in one way or another weave their way into people’s everyday activities, shaping the social world as it happens in a particular location (Smith, 1987, 1990b, 2005)

Because ruling in contemporary society is predominantly channeled via texts and documents, texts are essential to an institutional ethnographic inquiry. Smith (2005) articulates the interconnectedness of ruling, texts, and human action, she writes,

Institutions exist in that strange magical realm in which social relations based on texts transform the local particularities of people, place and time into standardized, generalized, and, especially, translocal forms of coordination people’s activities. Texts perform at that key juncture between the local settings of people’s everyday worlds and the ruling relations. (p. 101)

In that sense, texts, in their various forms, function as binding elements, connecting people across time and space, coordinating what they do. A crucial premise in institutional ethnography is that ruling is relational and enacted. People participate in—and reproduce—the complexes of ruling by engaging in certain texts, discourses, ideologies, concepts, theories, and standards in their local setting (Smith, 1990a, 1990b, 2005) According to Smith (2005), Foucault’s notion of discourse is a central aspect of ruling relations, as it locates knowledge “externally to particular subjectivities as an order that imposes on and coerces them” (p. 17). Discourses are not confined to statements *about* something but understood as systems of meaning embedded within people’s everyday practice. In this conception of discourse, meanings and doings are interconnected and interactive—meanings shape what we do, and our doings shape the meanings of what we do (Foucault, 1972; Smith, 1987).

Institutional ethnography is a project that turns the sociological enterprise upside down, by reinstating the subject as the starting point of inquiry, yet proceeding beyond the experiences of the individuals in a particular setting and into the examination of the formation of these experiences (DeVault & McCoy, 2006). The objective is to discover the way things “are actually put together” and “how it works” (Smith, 2006, p. 1) from a concrete standpoint within an area of everyday life. It is important to note that a standpoint does not reflect a specific position within society such as gender, class, and race: Instead, it denotes a place to start the investigation in the “local settings of people’s everyday experience” (Smith, 2005, p. 49). For example, one study by McGibbon et al. (2010) begins with the experiences of nurses and illustrates how various aspects of nurses’ stress, thematized as “emotional distress; constancy of presence; burden of responsibility; negotiating hierarchical power; engaging in bodily caring; and being mothers, daughters, aunts and sisters” (p. 1357), are linked to particular modes of ruling.

In this study, I take the standpoint of physicians. This is a somewhat broad adaption, as the standpoint represents physicians in distinct professions who are positioned differently within the institutional setting, but whose work intersects or congregates around the referral document and the CPP policy. Hence, I follow the making and interpreting of the referral document as it pertains to the start of a CPP from the standpoint of physicians located at different points in the referral interchange.

### Data Collection and Materials

The study is part of a larger collaborative qualitative research project evaluating the implementation of CCPs in Norway. Jointly, the project explores how the CPPs are put into practice and experienced by patients, health care providers, and managers affected by the reform across four cancer pathways: lung, prostate, breast, and malign melanoma. Ethical approval was obtained from the Norwegian Center for Research Data (project number 58724). Semistructured interviews were conducted from May 2018 to January 2020.

The article builds on interviews with 12 GPs and 25 specialist physicians (*N* = 37) who have firsthand experience of working with CPPs. A combination of purposive and snowball sampling (MacDougall & Fudge, 2001) was used to ensure geographical variation and the inclusion of different groups of specialist physicians. The sample of specialists includes clinicians, surgeons, radiologists, nuclear radiologists, and administrative managers working across five hospitals, both local and university hospitals. All the informants received written information about the project prior to the interview. This information was repeated orally on the day of the interview before they signed the consent form.

We employed a combination of individual and group interviews. Four group interviews and one individual interview were conducted with GPs, whereas 23 interviews with specialist physicians were carried out as individual interviews. In two interviews, the specialist physician was accompanied by an administrator colleague. The author participated in both individual and group interviews, conducting three interviews with specialist physicians alone and 10 together with a research team member. Other research team members conducted 12 interviews with specialist physicians. Furthermore, the author conducted three group interviews with GPs together with a research team member, whereas one group interview and one individual interview with GPs were carried out by other research team members. All interviews were recorded and transcribed verbatim, and the research team members were given access to all data materials. The author has developed the findings and analysis for this particular article.

### Analysis

Guided by the main principles of institutional ethnography, discovering the institutional aspects coordinating the informants’ doings remained essential throughout the analytical process (McCoy, 2006). Data collection and analysis occurred interrelatedly, directed by Smith’s (2005) notion of identifying a “problematic” to examine. A problematic is something in the informants’ accounts that the researcher finds puzzling, such as a tension between the different forms of knowledge drawn upon in everyday practice. The problematic is not necessarily experienced as a problem by the interviewed informants, because people’s ways of doing things are usually taken for granted (Rankin, 2017; Smith, 2005); however, it is precisely by making the taken-for-granted activities and experiences of people problematic that it is possible “to examine how these particular things happen as they do” (Campbell & Gregor, 2004, p. 47).

The entry point to CPPs arose as puzzling early in the investigation, as it became increasingly evident that starting a CPP is far more complicated than the standardized procedure outlined in the policies. Taking the starting point of CPPs as a problematic allowed the author to explore how the referral process works from various positions in the setting, spanning both primary and secondary care. Interviews were analyzed by labeling all the work connected to the starting point of CPPs as “referral work.” The analysis progressed by indexing the accounts related to referral work. Moreover, the author examined the data for disjunctures/small problematics (Rankin, 2017) between CPP guidelines and everyday practice that could help in illuminating the work involved in starting CPPs and tracing how these activities are coordinated.

## Findings

### Referral Work: At What Point Is a Reasonable Suspicion of Cancer Achieved?

**Figure 2.**
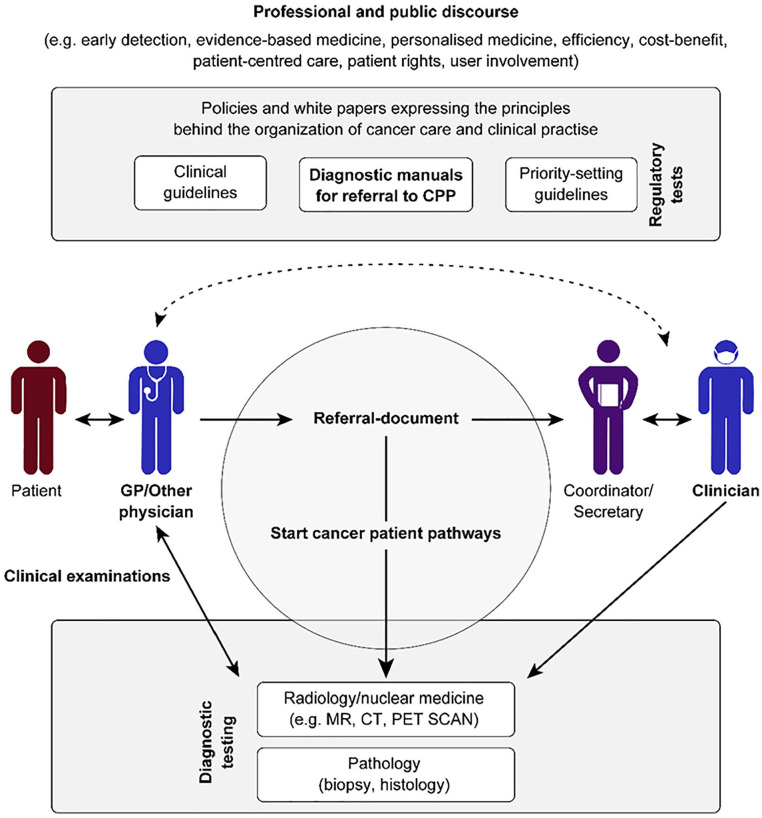
The figure illustrates that the starting point for CPPs relies on interaction between GPs and patients, as well as interaction between physicians in various locations in relation to the referral document. *Note.* CPP = cancer patient pathway; GP = general practitioner.

The findings are presented in two sections, first exploring the making and subsequently the interpretation of referrals, thereby following the natural order of things as they (for the most part) happen—that is, the move from primary to secondary care. Findings suggest that there are different interpretations of how the process of referring patients to a CPP is best realized, a key question being, at what point is a reasonable suspicion of cancer achieved? Physicians have different perceptions of how close to a final diagnosis a patient should be before it is appropriate to start a CPP. The findings also reveal some controversy over who in the specialist health services should be allowed to convert CPP referrals. Figure 2 establishes how the starting point of a CPP can lie in several interfaces between primary and secondary care, and how the work of starting a CPP is tangled within a complex set of relations that are discursively mediated. How these relations influence the referral process to a CPP is subsequently explored below. This is by no means an exhaustive outline, but it provides insight into some aspects of the social organization of referral work tied to the starting point of CPPs.

#### Managing patients’ worries and establishing a suspicion of cancer

GPs portray cancer as a challenging but common theme of daily practice. For some GPs, the CPPs are a welcome addition to practice because they have made the initial stages of cancer detection more of a shared responsibility between primary and secondary care physicians. CPP is described as a category that “makes things happen.” The referral process is said to run more smoothly as patients move quicker through the system. However, cancer diagnostics are also characterized as such a fear-inducing and complicated landscape to navigate that referring patients to a CPP is far from being a straightforward procedure.

##### Forming a suspicion of cancer: Moving between the concrete and the intangible

A GP can refer a patient to a CPP when there is a reasonable suspicion of cancer. To assist this process, the health authorities have developed diagnostic manuals containing the criteria by which to establish a reasonable suspicion of cancer (Norwegian Directorate of Health, 2019a). As the initial recognition of potential cancer symptoms usually happens through face-to-face communication with the patient, a significant part of the GP’s work, as informants describe, involves moving from patient-reported worries and symptoms to forming their own suspicion, ultimately determining whether the patient qualifies for a CPP.

When GPs discuss how they work to identify cancer symptoms, they draw on holistic and patient-centered perspectives (Schneider-Kamp & Askegaard, 2020). GPs express that the fear of cancer is so prominent in society that stress reduction is a significant aspect of discernment. Because patients differ, it is also important to align the approach with the individual needs of each patient. For instance, one GP explains how the patient’s mindset and attitude determine how he responds to a concerned patient:

If I doubt that the patient will follow up, because some [people] are so afraid to go to the doctor that they are like, “I finally got myself here, so things has to happen immediately otherwise I’m not going to bother doing anything about it,” and if I believe that it could be something serious I often “ok then, you are going [to the hospital] right away so I can be certain it gets checked, may not be anything concerning but at least we’ll know.” (GP, interview 5)

GPs emphasize the importance of forming a joint understanding between patient and doctor about the situation in question. This is done by evaluating the specific concerns conveyed by the patient in light of the patient’s medical history, which includes identifying the prevalence of cancer in the patient’s family. Many describe this as a tension-filled process, because cancer symptoms range from alarmingly clear to troublesomely uncertain. As one GP puts it,

It is a huge grey area with regards to, I mean, we move between rational and irrational thoughts, our worst fear is irritable bowel syndrome with loads of diffuse bowl problems and the day it turns out to be cancer we get hanged and we hang ourselves, and that is the chaos we live in, and actually get quite good at, but it is extremely stressful this particular field [cancer], and assessments are made in a kind of bewildering landscape (GP, interview 2).

Although all GPs confirm that they have access to the diagnostic manuals, they describe using them to a variable degree. One newly qualified GP comments, “I often check the criteria; when is it a suspicion, when should I refer, I try to follow it [the manual]” (GP, interview, 3). Others are more critical to guidelines explaining that there are so many guidelines for general practice that there is no way they can stay up to date on all the formal aspects related to the referral of patients. Furthermore, a strict adherence to guideline criteria is described as contradictory to how they practice:

No, actually, I don’t [look at guidelines] because there is no room for that, I have far too much to do to sit around and speculate over whether it checks five points or four points, I mean, I don’t give a crap. I’m thinking “Okay, this is urological cancer. I mean he pees blood, he is still peeing blood, I can’t find anything else, he needs to go in [for examination], it is cancer! Cancer Patient Pathways!” It is cancer, whether it checks five points or seven. I’m not engaging in that. (GP, interview 2)

It is hard, it is very vague what patients present, so let’s say the patient has lost weight or experience night sweats, and then they have to display all the symptoms required for CPP referral, that is not going to work, they might experience one symptom, or two, and it could be a full blown metastatic cancer. (GP, interview 4)

According to GPs, limited time combined with a mountain of guidelines for various types of referrals make it both challenging and somewhat irrelevant to practice a strict adherence to guidelines. Many GPs describe employing a combination of guidelines and intuition, as well as interaction with both the patient and colleagues, in forming their opinion. Much in line with the literature on professional discretion (Greenhalgh, 2002; Timmermans, 2005), informants describe the “gut feeling” as a kind of guiding compass, essential for tolerating the many uncertainties arising on a regular basis. A GP articulates it in the following manner:

And sometimes we makes mistakes, we have patients that both exaggerate and minimizes their symptoms, sometimes you take a blood test and can’t find anything, you just can’t make sense of it, but in most cases I have to trust that I have a kind of gut feeling for these things, otherwise I’m not going to be able to live with the uncertainty this job entails, and if the patient agrees, then I have a confirmation that I’m on the right track. (GP, interview 2)

A key point, underscored by GPs, is that cancer symptoms reveal themselves in such diverse and often vague ways that patients rarely match all the criteria for a CPP referral. The interactive context of establishing a suspicion becomes visible in the statement above. As patients report on their symptoms in different ways (some amplify whereas others downplay their situation), GPs make an active choice to trust their own instincts while searching for confirmation that they are on the right track in the feedback from the patient. Moreover, some GPs underscore that direct communication with hospital specialists is of greater value than formal guidelines because it is effective in sorting out misunderstandings and clarifying expectations. This echoes previous studies identifying the multitude of influences informing GPs’ referral practices (Gabbay & Le May, 2004; Greenhalgh, 2002; Nilsen et al., 2011; Thorsen et al., 2012).

##### “I’m to blame”: Balancing professional integrity and patients’ demands

The decision to refer is portrayed as being fraught with quandaries, specifically tied to an experience of having a dual responsibility. GPs explain that they are supposed to help patients access the care they want and feel they need, but they do not want to burden the system unnecessarily—a predicament portrayed in the literature as arising from GPs’ somewhat conflicting roles as both a patient advocate and a gatekeeper (Matthews, 2012). This friction in answerability organizes the GPs’ work in diverse ways.

Informants assert that their ability to filter patients successfully is reflective of their professionality in the broader community of physicians. This is exemplified below, where two GPs discuss how the lack of clarity over what level of uncertainty is acceptable in a CPP may compromise their reputation:

I was in so much doubt regarding a [female patient] that had stool changes and she had lost some weight; then we took supplementary blood tests that showed serious iron deficiency anemia and she was not that old, [an] otherwise healthy woman, so here I’m thinking “this is cancer till proven otherwise.” And then I began to doubt—is this enough for a CPP or is it somewhere in between? So, I ended up merely describing it . . . but I don’t know if the intention is there, that you are supposed to use CPP on those. What do you do? (GP, interview 1)

No, that is a grey area where you feel . . . I feel like it challenges my honor. I’m not keen on referring people for nonsense, and [in that case] you could say it is cancer till the opposite is proven, but at the same time, it may be just an innocent bleeding from the colon, so I don’t think I would refer that [to a CPP], but like you, describe it. (GP, interview 1)

What is striking about these statements is that the informants’ awareness of how others in the medical community “judge” baseless referrals enters the equation in such a way that it shapes the decision in favor of not activating a CPP, despite both GPs being convinced that the symptoms should be treated as a likely cancer. Informants explain that the medical community in Norway is so small that they often worry about their reputation as physicians. As one GP states, “you don’t want to be known as one of those GPs that constantly refers patients for cancer assessments and nothing is ever found” (GP, interview 3). And, some patients are more demanding than others. Balancing the needs of each patient with professional integrity is especially challenging when patients persistently refuse to accept the GP’s decision that no further testing is necessary. For example,

We get a lot of pressure from patients. There are so many instances supporting the patient, they have complaints . . . the [Health and Social Services] ombudsman, and they can complain everywhere really, but we sit by ourselves and then, for example, they want an MRI or a CT of their abdomen because they have a pain in their stomach. Bloodwork doesn’t show anything—most likely it is nothing. One in a thousand perhaps have something, cancer or something, and if you persist and deny the patient that CT because . . . or a PSA test, someone with prostate cancer, according to national guidelines there is no point in doing a PSA; suddenly someone gets prostate cancer and then it is us who are bashed in the media or other places. (GP, interview 4)

GPs assert that by being the first medical professional to see the patient, any detection of future illness may potentially be traced back to a past GP consultation. A patient who is denied access to diagnostic tests could at some point face serious consequences for their career. The informants emphasize the power of patients through public institutions such as the Health and Service Ombudsman (Helsenorge, 2019), whose primary mission is to safeguard patients’ interests. To prevent the possibility of patients launching formal complaints, they are willing to set aside their medical authority and follow instructions from the patient.

The extensive anxiety and uncertainties surrounding cancer symptoms makes balancing the aspects of gatekeeping and advocate quite challenging. Pressure from patients combined with concerns about missing a cancer and receiving complaints make it difficult to deny patients the testing they want despite the lack of obvious symptoms. One GP describes this pulling in different directions as a feeling of being held hostage:

. . . then I find myself in a dilemma that perhaps is more on an overarching level, and I agree, we tend to fire away, we over-examine and the carcinophobia out there makes us over-examine. Then I think, I sit down, take an anamnesis and monitor for three weeks, but I experience it as stressful that we have a responsibility not to [over-]examine, and that will cause us to miss slightly more of that weird, random stuff that an examination . . . and then, I don’t know if you guys feel the same way, but I’m to blame. I think it is both creepy and a bit unfair that I’m being given this hostage role, I’m not supposed to over-examine you, I’m not supposed to under-examine you, and I’m supposed to look after [you], and refer to CPPs, or I’m not supposed to, not too much, not too seldom, but if it goes wrong . . . I’m to blame. (GP, interview 2)

The GP describes feeling trapped by the conflicting demands of the institutional framework organizing the health care services. Ultimately, this becomes a question of compliance with a certain ideal, in this case, a responsibility not to overexamine, which is in line with the institutional discourse portraying excessive testing as a prevalent problem across the world (Bhardway, 2019; Brownlee et al., 2017). The account implies that the choice between leniency or restraint is one of damned if you do, damned if you do not. Choosing to incorporate a strict practice of restraint and hold back on these “just-to-make-sure” investigations requires a tolerance of uncertainty (Hoffman & Kanzaria, 2014). This includes the possibility of missing serious cases of illness, at least in their early stages, which policies such as the CPP are supposed to prevent.

##### Making the referral

The threshold for initiating a CPP varies between GPs. GPs distinguish between certain and uncertain cases and degrees of suspicion and explain that the decision to refer a patient to a CPP hinges on the level of doubt associated with the case. A definitive CPP referral is often described as “finding a lump in the breast.” However, most patients display far subtler symptoms requiring more extensive assessments. According to one GP, “you use it [CPP] when you are fairly certain, if you have a clear finding on a picture, for example an x-ray or some form of pathology blood test” (GP, interview 1). This is in line with another study (Jensen et al., 2014) showing that GPs may suspect cancer without initiating a CPP, and that patients whose symptoms are interpreted as “vague” are less likely to be referred to a CPP than patients with more telling symptoms.

Informants explain that the referral document is set up in a way that allows them to make concrete priority decisions through check boxes They may choose between multiple check boxes spanning 1 day to 4 weeks, and there is a separate check box for CPPs. Discussions on the relevance of crossing the time frame box reveal divergent understandings and practices; indeed, although some use them consistently, others say they never cross of the check boxes. A few GPs were not even aware that there is a CPP check box. GPs with experience of using the boxes underscore that the CPP box is the only box worth using as the other time frames in the referral are usually ignored by the receiving hospital.

However, the general agreement is that writing a precise text is the decisive factor. Several GPs explain that they usually write “cancer patient pathway” or “must be checked immediately” in capital letters to make sure that the referral does not slip through the cracks. It is vital that the referral adequately conveys how the patient’s symptoms relate to cancer. The way the text is written, and subsequently read, will determine how fast the patient will receive specialist health care.

#### Receipt of referral: Prioritizing by interpreting the need for urgency

A CPP starts the moment the hospital receives the referral. The first person to assess the referral in the hospital is usually a cancer pathway coordinator. The coordinator is responsible for scheduling the first appointment within CPP time frames and uses the referrals to ensure that appointments are distributed between patients according to priority, as indicated by the referrals. After assessing the referral, which sometimes includes marking it as CPP, the coordinator passes it on to the physician, who is ultimately responsible for determining whether the referral meets the requirements of a CPP. So, even though it lies in physicians’ domain to decide whether the referral should be categorized as a CPP, in some places, this work is actually done by the coordinators.

##### The diverse quality of referrals

Consistent with previous research, informants describe referrals as being “good, insufficient or bad” (Thorsen et al., 2013, p. 95). Some are explicitly marked CPP (by text or via the check box), whereas others only contain a description of the patient’s symptoms, therefore leaving it up to the specialist physician to judge whether it signals cancer. Informants explain that this is a problem because referrals marked CPP get “flagged in the system” and are tended to quicker than referrals that are not. Nonetheless, the recipient physician organizes the referrals according to CPP criteria; they must either mark referrals that match CPP criteria but where CPP is not initiated by the referring physician, or reject/deprioritize referrals that are marked CPP but where the description of symptoms does not qualify as a reasonable suspicion of cancer in their opinion.

One physician explains what it means to write a good referral:

That [GPs] have physically examined the patient. Before . . . [the patient] came in and stated that [they have] felt a lump on [their] right or left side and then the doctor would write “lump right breast” and send [the referral] off. Those get rejected. You have to conduct a clinical examination; how large is the finding, upwards, downwards, is it a hard lump, say something about the lump, do other family members have breast cancer? Because that is a criterion for determining whether they should enter the CPP or not. (Physician, hospital 3)

This illustrates the importance placed on distinguishing between patient-reported symptoms and a medically recognizable suspicion of cancer. The referral must document the medical practitioner’s suspicion of cancer as inferred from a physical examination and the patient’s history. Arguably, this shows how the referral process involves a meticulous distinction between “facts and fiction” for defining what kinds of experiences and signs warrant further investigation. Smith (1990b) underlines the social organization constituting facticity; she states that

it is the use of proper procedure for categorizing events which transforms them into facts . . . If something is to be construed as a fact, then it must be shown that proper procedures have been used to establish it as objectively known. (p. 27)

The categorizing of a patient’s experiences and bodily symptoms as cancerous requires a shift from the subjective to the objective, textualized, reality of what counts as symptoms of cancer.

The extent to which specialist physicians adhere to the CPP guidelines for inclusion varies. Although some say that they consistently reject insufficient referrals, others stress the importance of including patients in CPPs regardless, to avoid unnecessary delays for the patient. The uncertainties and possible errors that accompany the interpretation of cancer symptoms make it relevant to provide a speedy diagnostic trajectory for all patients that could potentially have cancer, even those with vague symptoms. A key argument is that CPPs work by bypassing ordinary waiting lists in the hospital. Consistent with a qualitative study on CPPs from Sweden (Delilovic et al., 2019), several informants deemed it likely that the introduction of CPPs has meant longer waiting times for patients who are not categorized as belonging to an urgent priority group. Delilovic et al. (2019) refer to this as the unintended “crowding out effects” (p. 6) of CPPs, which is defined as “situations where lower priority patients are given care before patients who have a higher priority” (p. 6). Arguably, this is highly significant. Longer waiting time imposed on patients outside of the CPP system could compromise the timeliness of care to those patients who are seriously ill but where this is not adequately conveyed by the referral, or who are considered a lower priority by the specialist than the referring physician intended.

##### Negotiating priority settings

One point of contention (and frustration) raised by physicians working in imaging departments concerns their lack of rights to reject or downgrade what they refer to as erroneous CPP referrals. In principle, they are not allowed to convert CPP referrals, which becomes a problem because of the variable quality of referrals and the limited appointments available. Several informants express that this an ongoing topic of debate. For example,

We are also responsible for the CPP with serious, [unspecific] symptoms, and I have to say that, in my experience, it’s been quite misused, at least in the beginning. I mean, “we want a CT fast, so we just mark it CPP with serious symptoms,” and when you read the referral it doesn’t really fit the criteria, but it is stamped CPP so . . . I’ve tried to send them back, but then it became a topic of discussion at the top level, above me, and it was decided that I couldn’t do that, so we just have to run [the tests as CPPs] and describe them. (Physician, hospital 2)

The physician notes that there is an inconsistency between guideline criteria and actual practice. He speculates that physicians are using CPPs as a means to secure their patients faster assessments. He also experienced that his professional judgment was overruled by the managers “at the top level.” When asked why they are not allowed to alter CPP referrals, informants assume that it is part of the policy. Similarly, others argue for the opportunity to convert CPP referrals, because they embrace such a wide variety of symptoms, from relatively low to high suspicion of cancer, that it defies professional logic to place them all in the same priority category.

However, some say that in their department, the resources are so scarce that they have no choice but to downgrade some of the CPPs to make sure that the patients “that need it the most” are dealt with first. For example,

I don’t place all the CPPs at the top—I look for medical indications. For example, lymphoma is urgent. A lymphoma in a 20-year-old is more urgent than an 80-year-old prostate cancer patient, because prostate has a slower progression rate than lymphoma. Lymphoma can kill within a month. (Physician, hospital 3)

The physician explains that she organizes referrals according to medical indications and the characteristics of the patients, as outlined by clinical practice guidelines, rather than the standardized time frames suggested by the CPPs. Clinical practice guidelines provide evidence-based recommendations for decision making related to diagnosis and treatment (Timmermans, 2005). This suggests that by activating another set of guidelines, physicians are able to prioritize referrals differently and in a less standardized way than allowed for by the CPPs. This is deemed necessary because the level of urgency is different between potential cancer patients, even those with the same type of cancer.

One radiologist physician specializing in breast cancer describes how they have solved this predicament in her department:

Physician: It is very frustrating because the hours [appointments] are limited. So, in the beginning we used to call the GP and explain that this is not good enough, we want a new referral, but now we have found a way around it, so we don’t do that anymore. We downgrade the CPPs even if we are not allowed to, but then we put it on a list and that list is handled by the secretary. They send a letter to the GP, informing that [the referral] does not fit the criteria and that the GP is free to contact us if [s]he disagrees. It makes it better for the patient because we downgrade it right away in order to give the patient an appointment immediately. Before, we would wait for the next referral, and that caused some delays.

Interviewer: So that means that the patient gets an appointment anyway?

Physician: Gets an appointment, but not within the CPP timeframe, right? Perhaps we consider it to be a lump in the breast that needs to be addressed within four weeks, so she [the patient] gets an appointment within that deadline. (Physician, hospital 3)

This reveals an interesting aspect of the CPPs’ time frames—that they are not based on medical indications and are thus not legally binding. Consequently, it is not a patient’s right to access specialist care within the CPP time frames, even if the referral is classified as a CPP (Norwegian Directorate of Health, 2019b). The other deadline referred to in the statement is anchored in the priority-setting guidelines, which were introduced prior to the CPPs (from 2008–2012) to align professional discretion better with the overarching political and judicial principles for the prioritization of health care services (Aase-Kvåle et al., 2019). These guidelines specify which conditions give patients the right to specialist care and provide recommendations for the maximum deadlines to start treatment (Tranvåg et al., 2015). By introducing the CPP policy, medical professionals must now juggle two types of deadlines anchored in different policies. An important difference between the two lies in the deadline for assessing referrals; according to the priority-setting guidelines, referrals must be assessed within 10 days, whereas many CPPs require that the patient meets with a specialist physician within 7 days (Norwegian Directorate of Health, 2016, 2019c).

The physician explains how she negotiates between these two policies when she prioritizes referrals for potential breast cancer:

Physician: We must respect [the legal deadline] if there is a lump, even if it appears quite innocent, but we do make an assessment of malignancy potential. So, we distinguish between those that the GP has felt “this one is scary,” so they can come straight in, right? A new, unexplained irregular lump with contracture, right? That is highly suspicious.

Interviewer: And you can feel that just by touching?

Physician: A GP will be able to feel that. But, of course, mistakes are made all the time, but [patients] are protected by that four-week [deadline] . . . because if we fill all our CPP appointments with things that aren’t important, the entire CPP program will fall apart. (Physician, hospital 3)

This is particularly interesting as all the GPs describe a lump in the breast as undoubtedly a CPP because all lumps are potentially malignant. However, for the radiologist, that is not the case: All lumps should be checked, but not all lumps have the same urgency and they must be prioritized accordingly. In doing so, she uses the medical indications conveyed by the referral to differentiate between referrals according to the criteria outlined by different guidelines.

Apparently, decisions about priority are based on a more fine-tuned medical distinction by specialists than by GPs. However, in discussing the varying quality of referrals and priority settings, it is critical to note that most specialist physicians assert that they understand the difficult position of the GP. They easily imagine that GPs are pressured by patients who are anxious despite a low medical indication that there is anything malignant to worry about. Clearly, it is a significantly different procedure to sit face-to-face with a concerned patient than it is to categorize a document. For the specialist, the referral document is a representation of the patient, and thus the closer the referral reflects the reality of the patient’s situation, the easier it is for the specialist to prioritize incoming referrals appropriately.

## Discussion

Guided by institutional ethnography, I have investigated the social organization of referral work in the context of CPPs. I discovered that the referral work involved in starting CPPs is complex, with several overlapping interfaces between primary care and different hospital departments. An important analytical point in this study is that “the way things happen as they do” (Campbell & Gregor, 2004, p. 47) depends on the way people interact with the conditions of their practice (Smith, 1990b). The gateway to CPPs relies upon the interaction between physicians and patients and how the referral is composed, as well as how and by whom the referral is interpreted. The inquiry revealed that the distinction between CPP or not is by no means clear-cut for either primary or specialist physicians. Furthermore, the findings have illuminated some of the policies and discourses that mediate the work of starting a CPP.

GPs assert that fear and vague symptoms are major triggers for the frequent concerns about cancer being raised by patients. The ambiguous nature of cancer symptoms makes it challenging to navigate the border between rational and irrational concerns from a medical point of view. Therefore, GPs invest significant energy in assessing the patient, employing a patient-centered approach upon referral. This is in line with the extant literature (Gabbay & Le May, 2004; Greenhalgh, 2002; Thorsen et al., 2012; Timmermans, 2005), noting that the choice of whether to refer a patient is regulated by a combination of the physician’s experience, professional judgment, and collegial relations; the patient’s subjective concerns and experiences—how these are conveyed by the patient and interpreted by the physician; and official guidelines for practice.

GPs’ work is organized by ruling relations driven by competing interests, whereby the GPs become responsible both for protecting the system’s capacity and helping patients to access specialist care. Occasionally—or perhaps, more specifically, when vague symptoms intersect with what they refer to as demanding patients—these interests create a dilemma wherein the GPs must negotiate between the patient’s desire for diagnostic testing and their own professional integrity. Interestingly, the practice of restraint—for example, waiting to see how the symptoms develop—is tied to a sense of honor in the wider community of physicians, whereas the practice of leniency is tied to patient satisfaction and the power of patients to launch formal and informal complaints. Either way, the GP’s reputation is at stake.

The GPs have different interpretations of when it is appropriate to initiate a CPP. Some refrain from using CPPs in cases where they suspect it might be cancer but do not feel the symptoms can be clearly defined, whereas others see the CPP as an obvious choice in uncertain cases. Because of the GPs’ various thresholds for enacting the CPP, some patients may wait longer than others to be referred. In addition, the amount of diagnostic testing performed by GPs prior to patients entering a CPP varies, which means that patients could be at very different stages in the diagnostic process upon entry to a CPP. This, of course, will greatly influence the pace of the entire CPP trajectory.

As with the making of referrals, several tensions between institutional policies and actual work practices come into play in the work of interpreting referrals. These tensions revolve around the diverse quality of referrals, physicians’ knowledge of the complicated nature of cancerous diseases, and the freedom (or lack thereof) to apply one’s own professional discretion. This work is embedded in the principles for prioritization by which the specialist health care services are organized more generally, as well as cancer care more specifically (Norwegian Directorate of Health, 2019b, 2019c; Norwegian Ministry of Health and Care Services, 2017).

Prioritizing is a natural and ingrained part of the daily work of specialist physicians. In Norway, the authorities have discussed questions pertaining to priorities in health care for more than 30 years; this has resulted in the successive development of policies establishing the premises on which patients should be prioritized (Norwegian Ministry of Health and Care Services, 2016). The CPP policy is thus part of a broader discourse on prioritization in public health care. By specifically targeting potential cancer patients, CPPs enable these patients to bypass the regular hospital waiting lists, which are organized according to another set of priority-setting guidelines. These other guidelines are anchored in the more overarching policies specifying that prioritization between patients is supposed to happen based on the degree of seriousness and need for urgency. This involves a decline in prognosis with regard to life span and quality of life if help is postponed (Bjorvatn & Nilssen, 2018).

The intention of CPPs is to provide every person with a reasonable suspicion of cancer the same care package within a standardized time frame (Norwegian Directorate of Health, 2016). This implies that, in principle, all CPP patients belong to the same priority category. However, the diverse quality of referrals makes the classification and prioritization of patients according to CPP criteria challenging, and physicians engage with referrals in different ways. Ultimately, they use a combination of professional discretion and different guidelines to negotiate prioritization between patients.

## Conclusion

As previously mentioned, the scope of this study is too narrow to detail all elements of the social organization that influence the starting point of CPPs. Rather, it highlights some aspects of the ruling apparatus that mediate the experiences of physicians. A limitation connected to the methodological framework is that this study is part of a wider collaborative project and the interviews were conducted by different researchers. Although all the interviewers used the same interview guide, other researchers did not proceed with an institutional ethnographic perspective in mind, and so opportunities to explore traces of ruling relations as they emerged during interviews could have been missed. Also, this study is conducted in a Norwegian context, focusing on experiences tied to modes of ruling particular to this society. However, as many countries have introduced similar reforms and guidelines, the findings may be relevant for other health care systems.

The findings of this study have important implications for further development of the CPPs. Although equal rights to a fast-track care trajectory is a great ambition that is impossible to disagree with, it is evident that not all potential cancer patients can or should be treated as belonging to one single group. A key point is that cancer is detected in various stages of development, and thus it could be argued that the standardization of time frames for diagnosis and treatment in a “one-package-fits-all”–type model such as the CPPs discredits physicians’ professional authority. Furthermore, it portrays rapidity within the health care services as synonymous with high-quality care, an image consistently disputed by our informants, who argue, from a patient-centered perspective, that patients have diverse needs and desires, which must always form part of the equation.
